# Investigation of an algae-derived polymer as a pollinator-friendly pesticide adjuvant

**DOI:** 10.1038/s41598-025-10558-1

**Published:** 2025-07-15

**Authors:** Narayanan Kannan, Yu-Cheng Zhu

**Affiliations:** USDA-ARS-Pollinator Health in Southern Crop Ecosystems Research Unit, Stoneville, MS 38776 USA

**Keywords:** Insecticide, Honeybee, Spray, Drift, Toxicity, Bioassay, Ecology, Environmental sciences

## Abstract

Chemical applications in croplands can be a major source of pesticide exposure to pollinators, including off-target drift. Adjuvants are added to pesticides to improve spray characteristics. The major goal of this study was to identify the possibility of using sodium alginate (SA) as a drift-reducing pesticide adjuvant that is less/nontoxic to honeybees than polyacrylamide (PAM) an industry standard. The toxicity bioassay experiments were carried out with six- to eight-day-old, caged honeybee workers. Each cage contained 20 honeybees. Caged bees were treated with adjuvant, insecticide, adjuvant + insecticide, and a water only as a control, using a modified Potter Spray Tower. The insecticides imidacloprid and bifenthrin were used at three different concentrations (LC_25_, LC_50_, and LC_75_). Two different concentrations of the adjuvants SA and PAM were tested. The 48-hour mortality results were recorded. Our results indicate noticeable reductions in bee killing when SA is used as an insecticide adjuvant instead of PAM.

**Supplementary Information:**

The online version contains supplementary material available at 10.1038/s41598-025-10558-1.

## Introduction

The lower Mississippi Delta (LMD) is a very productive agricultural region in the USA. Soybean, cotton, and corn are the major crops cultivated (USDA-NASS 2017). *Lygus lineolaris* (tarnished plant bug) and *Piezodorus guildinii* (red-banded stink bug) cause significant damage to cotton and soybean in the region. Insecticides are used to control crop pests to enable profitable commercial-scale crop production. The beekeeping industry is also booming in the region^1^. However, the health of bees raised in areas adjacent to cropped areas is affected by pesticide exposure, including off-target drift^2–7^. In addition to the pesticide active ingredient that targets the pest (insect/weed/fungus), several other chemicals (called adjuvants) are added to make the pesticide mixture in the tank more efficient to apply. The functionalities of adjuvants include a reduction in off-target pesticide drift, the ability of pesticides to stick more to the target surface, facilitating absorption^8,9^penetration of pesticides through the target surface^10^and translaminar redistribution and systemic movement within plants^11^.

There is some evidence in the literature that adjuvants added to insecticides are equally/more toxic than the active ingredient^12–17^. One such adjuvant is polyacrylamide (PAM). Previous research has shown that high levels of acrylamide cause cancer in laboratory animals and are reasonably anticipated to be a human carcinogen^18,19^although the risk that acrylamide poses to humans is not clear. However, the United States Food and Drug Administration (USFDA) has prepared guidelines for food industries to minimize the exposure of acrylamides^20^.

The aggravated toxicity of pesticide tank mixtures^15,21^ must be minimal to humans and nontarget animals, especially pollinators (such as honeybees), which play an important role in our food production system. Any adjuvant that is compatible with commonly used herbicides and insecticides with no interference with pesticide intoxication mechanisms and no aggravated toxicity on beneficial insects such as bees and other pollinators will have a very large positive effect on the pesticide market. Therefore, we attempted to develop a pesticide additive that acts as an adjuvant to apply pesticides to the target surface, mitigates off-target drift and has non/less toxicity to beneficial organisms such as pollinators. The proposed adjuvant material is sodium alginate (SA), an inexpensive natural linear polysaccharide commercially produced from brown algae (Phaeophyceae, one of the major seaweeds of temperate and polar regions)^22^.

Our prior experiments in the laboratory and field demonstrated (a) the drift reduction potential of SA for ground^23^ and drone-based aerial applications^24^ and (b) its synergistic activity with insecticides in killing *L. lineolaris* (tarnished plant bug) and *P. guildinii* (red-banded stink bug)^25^. The toxicity of SA to honeybees is not known. Therefore, the main goal of this study was to analyze the applicability of SA as a pollinator-friendly insecticide adjuvant. The specific objectives include performing bioassay experiments with six- to eight-day-old honeybee workers to identify any changes in bee survival as an effect of any synergistic/antagonistic effects of the adjuvant on the insecticides.

## Materials and methods

### Pesticide adjuvants

Sodium alginate (SA) is a nontoxic, biocompatible, biodegradable^26^and water-soluble polymer. The SA is listed under the US FDA as a “generally recognized as safe” (GRAS) material. In the European Union (EU), it is registered as a food improvement agent^27^. The United States Environmental Protection Agency (USEPA) rated SA as a safer chemical^28^. SA is used in drug delivery systems and as a wound dressing material in the medical field^29–31^; as a thickener in the food industry; and as a texturizer, stabilizer, firming agent, flavor adjuvant, surface active agent, processing aid, emulsifier, flocculant in water treatment, drilling mud and formulation aid^32^.

One of the common active ingredients in existing industry standard pesticide adjuvants is polyacrylamide (PAM). PAM is a nonionic, water soluble, and biocompatible polymer. It is available in both solid and liquid forms. The largest use of PAM is in the water and wastewater treatment industry as a flocculant^33,34^. It is extensively used in soil conditioning and erosion control^35–37^ and in the oil and gas industry^38^.

### Physicochemical characteristics of the adjuvants

Millipore pure water was used as a reference to compare the physicochemical characteristics of the adjuvant solutions at different concentrations. A pH meter (Thermo Scientific, Chelmsford, MA, USA), calibrated with a standard solution with a pH of 7, was used to measure the pH values of the test solutions. The density was measured using a specific gravity hydrometer (VWR, Radnor, PA, USA). All the pH, density and dynamic surface tension measurements were carried out at 23 °C. The viscosity values were measured via an IKA Rotavisc lo-vis control viscometer (IKA-Werke GmbH & Co. KG, Staufen, Germany) with spindle SP1 at 22 °C. The viscometer was calibrated with certified standard silicone oil. A KRÜSS bubble pressure tensiometer BPT100 (KRÜSS GmbH, Hamburg, Germany) was used to estimate the dynamic surface tension values of the adjuvant solutions and water. All the estimated physicochemical properties of the adjuvant solutions are presented in Table 1.

**Table 1:** Physicochemical characteristics of water and sodium alginate solutions.

Item	Appearance	pH	Density (g/cm^3^)	Dynamic Viscosity (mPas)	Dynamic Surface tension (mN/m)^#^
Millipore pure water	Clear liquid	7.00	0.9968	3.47	75.3–74.9
Sodium alginate 1.25 g/L	Nearly a clear solution	6.86	0.9968	5.70	76.0–75.0
Sodium alginate 2.5 g/L	Nearly a clear solution	6.92	0.9971	7.53	76.0–75.0
Polyacrylamide 75 µg/L	Milky suspension	5.83	0.9969	3.93	74.8–70.1
Polyacrylamide 187 µg/L	Milky suspension	5.99	0.9970	4.33	74.9–62.8

# Values represent surface ages of 10 milliseconds (ms) to 100000 ms.

### Insecticides

The formulated-grade insecticides used in this study were Advise Four (imidacloprid, 40.4%, Winfield Solutions LLC) and Tundra ^®^EC (bifenthrin, 25.1%, Winfield Solution LLC), which were purchased from local agricultural chemical suppliers near Stoneville, Mississippi, USA. The insecticides imidacloprid and bifenthrin were selected to represent the neonicotinoid and pyrethroid classes of insecticides, respectively.

### Preparation of honeybees for the experiment

Approximately 1,300 bees were obtained for the experiments. Deep frames with more than 50% coverage of healthy and sealed broods were pulled from 5 to 7 strong colonies and transferred to a lidded container vented by large cutouts covered by a mesh screen. The container was kept in a laboratory incubator (33 ± 0.5 °C; 60% ± 3% relative humidity; no light). Newly emerged worker bees were transferred daily in groups of 20 into each cage made from a 500-ml wide-mouth polypropylene jar (D×H: 9.3 × 10 cm) with a (3 × 8 cm) section of plastic foundation attached vertically to the inner side of the cage. Each of these cages had an 8.9 cm diameter (d) hole cut in the lid and covered with a 3 × 3 mm-mesh metal screen to prevent escape. Caged bees were provided with one scintillation vial (20 ml) of 50% (w/v) sucrose solution and one vial (20 ml) of water placed upside down on the top of each cage. Two holes (1.6 mm) were drilled in each vial cap. Caged bees were maintained in incubators for 6‒7 days before testing. Cages with more than five dead bees were not used for experimentation. Before the experiment started, dead bees were counted, and excluded from the total number of bees tested in each replicate.

The age of the honeybees used for this experiment was selected on the basis of our prior study, which revealed that 3-8-day-old bees have similar tolerances to insecticides^39^. When worker bees feed exclusively on a carbohydrate diet with no influence of a queen pheromone, they behave like foragers do^40^. On the basis of this premise, the bee diet for this experiment was formulated. Colony level differences in bees are dynamic, seasonal, and not manipulatable. Therefore, in this study, showing difference in results because of colony level changes in bees were not attempted. In summary, our laboratory setting/testing system closely simulates field spray exposure in honeybees.

### Spray equipment and spray solutions

A modified Potter Spray Tower (Burkard Scientific Ltd., Uxbridge, U.K.) was used to spray the solutions. Each 500 µL spray solution was delivered into a cage (replicate holding 20 bees) at 69 kPa (10 psi) from a spray distance of 22 cm in a controlled environment^15^. Deionized water was used to make the spray solutions at the desired concentrations. For each insecticide, three different concentrations representing the LC_25_, LC_50_, and LC_75_ values^15^ were used in the experiment. For each adjuvant, two different concentrations were used. The concentrations of 75 µg/L and 150 µg/L for PAM and 1.25 g/L and 2.5 g/L for SA were chosen based on laboratory and field experiments to minimize off-target spray drift.

### Bioassay experimental setup

Bioassay experiments were carried out with six- to eight-day-old honeybee workers. A water-only spray was used as a control in both experiments. Each replicate consisted of one cage of 20 honeybees that were six to eight days old. Three replicates were used for each treatment. A total of 63 cages were used to test each pesticide adjuvant. The number of bees that died after 48 h was used as the result of each bioassay experiment. The corrected mortality values were obtained via Abbott’s formula^41^ (Eq. 1).

1 $$\:{C}_{mortali}=100\times\:\frac{({mortali}_{TR}-{mortali}_{C})}{(100-{mortali}_{C})}$$

where $\:{C}_{mortali}\:$ represents the corrected mortality for a treatment and replicate mortali_TR_ is the 48-hour bee mortality for a treatment and replicate mortali_C_ is the average (of 3 replicates) 48-hour bee mortality for the control.

### Statistical analysis of bioassay results

SAS (version 9.4) software^44^ was used to compare significant differences among/between chemical treatments. Analysis of variance (ANOVA) and the Proc GLM (general linear model) procedure were applied with the option of Fisher’s least significant difference (LSD) method for mean separation at *p* = 0.05. To confirm the synergistic or additive interaction of adjuvants with insecticides, additional t tests were applied to reveal significant differences between the combined mortality of two separate chemical treatments and the mortality of mixtures of the same two chemicals. Two-sample t tests were carried out separately for mortality resulting from imidacloprid and bifenthrin. For each pesticide, the corrected mortality results from the control, adjuvant alone and pesticide alone treatments were also included. The null hypothesis (H_0_) was that “the mean values of the corrected mortality results for both adjuvants are not different”. The alternate hypothesis was (H_A_) “The mean values of the corrected mortality results for both adjuvants are different”. The acceptance/rejection of the null hypothesis was carried out based on the probability of the test results.

## Results and discussion

**Fig. 1:**
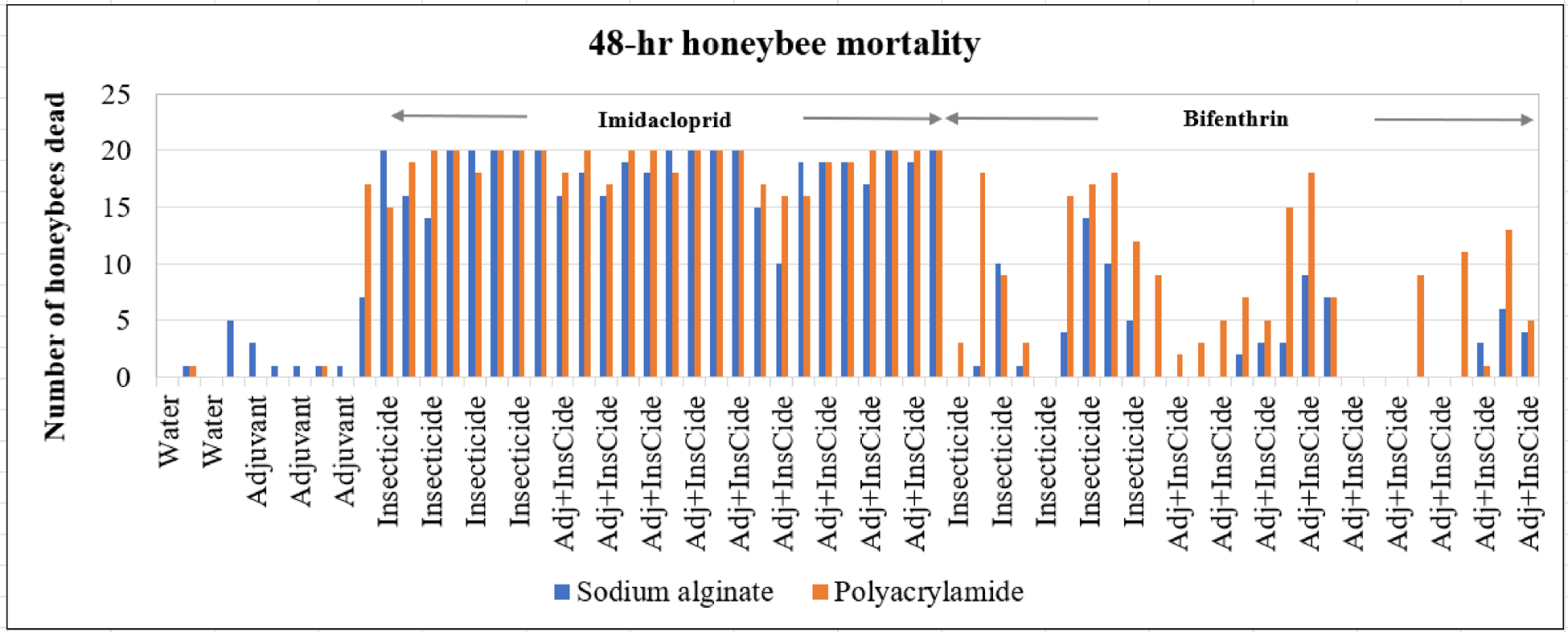
Forty-eight-hour honeybee mortality results after the toxicity experiment.

**Fig. 2:**
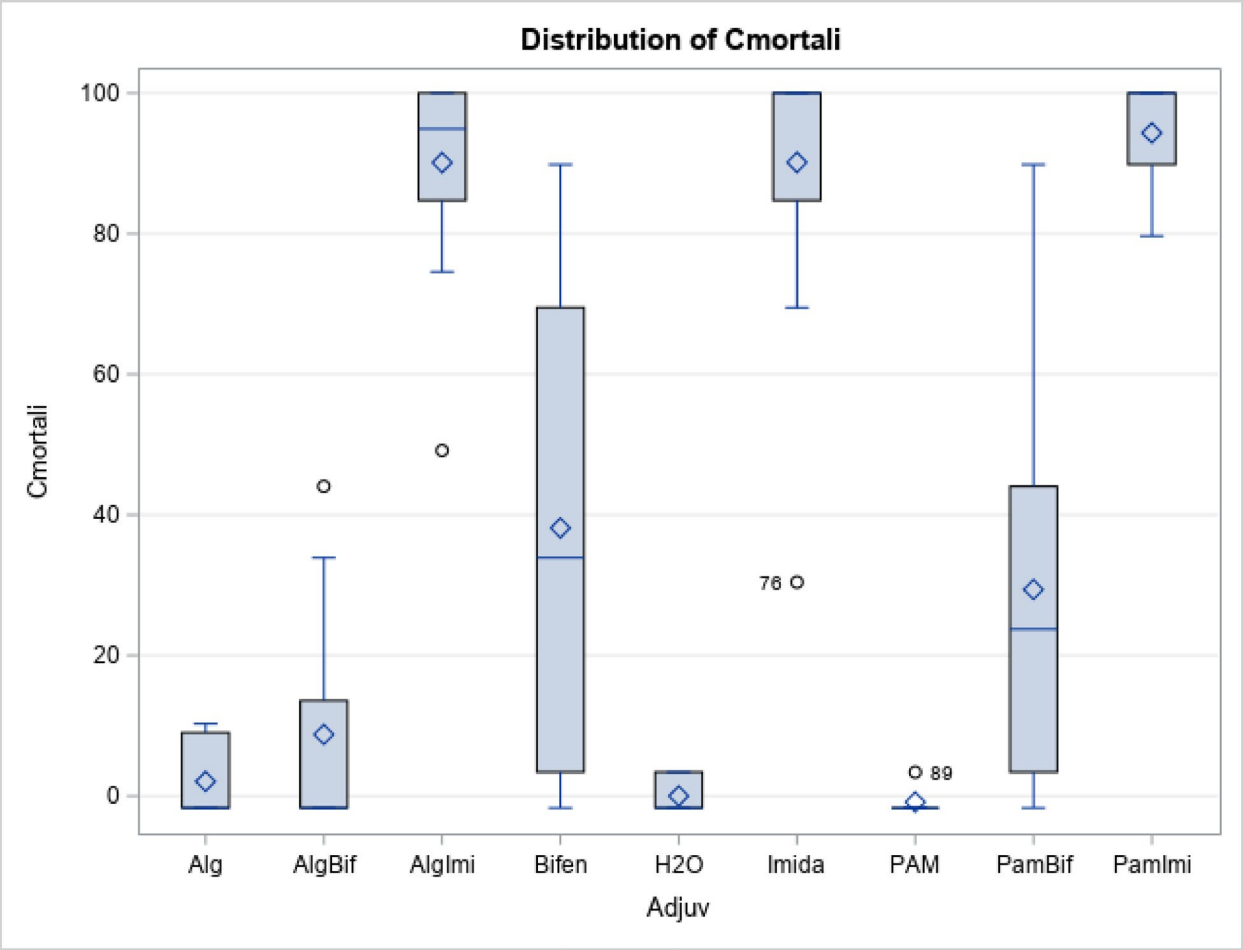
Box and whisker plot showing the distribution of the corrected mortality for different treatments (R^2^: 0.83 CV:36.1 Corrected mortality mean: 50.8) (Note: The middle of the diamond indicates mean, the top and bottom portion of the diamond indicate upper and lower confidence limits of mean, the line in the middle of the box indicates median, the upper and lower end of the box show 75th and 25th percentile, the data points outside the top/bottom whisker denote outliers in the data).

The raw data showing observations of 48-hour bee mortality after the toxicity experiment are presented in Fig. 1. The average corrected mortality for each treatment was calculated via Eq. (1) and is presented in Tables 2 and 3. A summary of the corrected mortality data is provided in Fig. 2 and Table S1 (supplementary material). Results of t test (LSD) for corrected mortality is provided in Table S2 (supplementary material) and the summary statistics for GLM procedure in Table S3.

**Table 2:** Average corrected mortality for different treatments involving polyacrylamide.

Treatment ID	Treatment description	Average corrected mortality
H_2_O	Water only	0.00
P75	75 µg polyacrylamide per liter of water	-1.70
P150	150 µg polyacrylamide per liter of water	0.00
I158	158 mg imidacloprid per liter of water	84.75
I276	276 mg imidacloprid per liter of water	96.61
I483	483 mg imidacloprid per liter of water	100.00
P75 + I158	75 µg polyacrylamide & 158 mg imidacloprid per L of water	91.53
P75 + I276	75 µg polyacrylamide & 276 mg imidacloprid per L of water	96.61
P75 + I483	75 µg polyacrylamide & 483 mg imidacloprid per L of water	100.00
P150 + I158	150 µg polyacrylamide & 158 mg imidacloprid per L of water	81.36
P150 + I276	150 µg polyacrylamide & 276 mg imidacloprid per L of water	96.61
P150 + I483	150 µg polyacrylamide & 483 mg imidacloprid per L of water	100.00
B150	150 mg bifenthrin per liter of water	49.15
B258	258 mg bifenthrin per liter of water	30.51
B446	446 mg bifenthrin per liter of water	77.97
P75 + B150	75 µg polyacrylamide & 150 mg bifenthrin per liter of water	22.03
P75 + B258	75 µg polyacrylamide & 258 mg bifenthrin per liter of water	27.12
P75 + B446	75 µg polyacrylamide & 446 mg bifenthrin per liter of water	66.10
P150 + B150	150 µg polyacrylamide & 150 mg bifenthrin per liter of water	-1.70
P150 + B258	150 µg polyacrylamide & 258 mg bifenthrin per liter of water	32.20
P150 + B446	150 µg polyacrylamide & 446 mg bifenthrin per liter of water	30.51

**Table 3:** Average corrected mortality for different treatments involving sodium alginate.

Treatment ID	Treatment description	Average corrected mortality
H_2_O	Water only	0.00
A1.25	1.25 g sodium alginate per liter of water	1.96
A2.5	2.5 g sodium alginate per liter of water	0.00
I 158	158 mg imidacloprid per liter of water	63.16
I 276	276 mg imidacloprid per liter of water	88.33
I 483	483 mg imidacloprid per liter of water	100.00
A1.25 + I158	1.25 g sodium alginate & 158 mg imidacloprid per L of water	80.00
A1.25 + I276	1.25 g sodium alginate & 276 mg imidacloprid per L of water	91.67
A1.25 + I483	1.25 g sodium alginate & 483 mg imidacloprid per L of water	100.00
A2.5 + I158	2.5 g sodium alginate & 158 mg imidacloprid per L of water	60.00
A2.5 + I276	2.5 g sodium alginate & 276 mg imidacloprid per L of water	88.33
A2.5 + I483	2.5 g sodium alginate & 483 mg imidacloprid per L of water	98.33
B150	150 mg bifenthrin per liter of water	18.33
B258	258 mg bifenthrin per liter of water	8.33
B446	446 mg bifenthrin per liter of water	46.67
A1.25 + B150	1.25 g sodium alginate & 150 mg bifenthrin per liter of water	0.00
A1.25 + B258	1.25 g sodium alginate & 258 mg bifenthrin per liter of water	8.33
A1.25 + B446	1.25 g sodium alginate & 446 mg bifenthrin per liter of water	31.67
A2.5 + B150	2.5 g sodium alginate & 150 mg bifenthrin per liter of water	0.00
A2.5 + B258	2.5 g sodium alginate & 258 mg bifenthrin per liter of water	0.00
A2.5 + B446	2.5 g sodium alginate & 446 mg bifenthrin per liter of water	20.00

Water alone control, and adjuvant alone (PAM 150, and Alg 2.5) treatments did not kill the honeybees. Alg 1.25 treatment killed about 2% of the bees. The imidacloprid-only treatment (I) killed almost all the bees. Similar results were obtained for the adjuvant and insecticide treatments involving both SA + I and PAM + I (Figs. 1 and 2; Tables 2 and 3). As a neonicotinoid insecticide, imidacloprid can kill all bees, although they can acquire some physiological resistance through metabolic detoxification^42,43^.

Imidacloprid is a synthetic neonicotinoid systemic insecticide used to control several sucking and mining pests. When sprayed, it is absorbed by plants through their leaves or roots and spreads throughout the plant. When insects eat treated plants, they also consume imidacloprid, which binds to the nicotinic acetylcholine receptor on nerve cells. This prevents acetylcholine from transmitting impulses between nerves, which leads to paralysis and eventual death of the insect^45^. The mode of action of imidacloprid on beneficial insects such as honeybees is similar to that of other insects^46^. The high 48-h mortality rates for honeybees observed in this study are justifiable and compare well with those reported in the literature^47^. Additionally, high mortality rates appear to be unaffected by the source/location of honeybees, as noted by Nauen et al. 2001^48^.

The bifenthrin-only treatment killed a significant proportion of the bees. However, there was some reduction in bee killing in the bifenthrin and SA treatments compared with the bifenthrin and PAM treatments (Figs. 1 and 2; Tables 2 and 3). Pyrethroids are human-made versions of pyrethrins derived from chrysanthemum flowers. Bifenthrin is an important pesticide from the pyrethroid family. When consumed orally or in contact, it interferes with the nervous system of insects^49^paralyzes the insect’s central nervous system, preventing it from eating or functioning. The relatively low toxicity of bifenthrin (compared with that of imidacloprid) observed in this study aligns well with what has been reported in the literature^50,51^.

The relatively lower mortality of bifenthrin with SA than with PAM is also clearly visible from the t test results (Table 4). The corrected mortality rates were lower for bifenthrin when SA was used as the adjuvant (mean 10.6%) than when PAM was used as an adjuvant (mean 27.7%) (Table 4). The differences are also clear from the variances (Table 4) and the probability value (0.069), although the probability was not below 0.05, the assumed level of significance (Table 5). For the pesticide imidacloprid, the t test results were not statistically significant, and the corrected mortality results were similar for both PAM and SA (Table 4 and Table 5).

**Table 4:** T test results: means and variances.

Results of t tests	Imidacloprid		Bifenthrin	
	PAM	SA	PAM	SA
Mean	70.5	67.0	27.7	10.6
Variance	1870.3	1664.2	708.5	241.7

**Table 5:** T test results: statistics.

Results of t tests	Imidacloprid	Bifenthrin
Degrees of freedom	22	22
t-Statistic	0.201	1.913
t Critical two-tail	2.074	2.074
P(T < = t) two tail	0.842	0.069

The concentrations of imidacloprid and bifenthrin used in this study were based on 2014 data^15^. During the last 10 years, there were certain changes or updates in pesticide manufacturers and formulations that may have influenced honeybee sensitivities to imidacloprid and bifentherin, as observed in this study. Furthermore, the use of bifenthrin has substantially increased, whereas the use of imidacloprid has significantly decreased nationwide during the past 10 years (https://water.usgs.gov/nawqa/pnsp/usage/maps/compound_listing.php). In insects (including honeybees), resistance/tolerance to insecticides is closely associated with insecticide exposure or selection pressures^52^. This also explains the relatively low mortality of honeybees with the insecticide bifenthrin compared with imidacloprid.

Within the bifenthrin treatments, the addition of SA as an insecticide adjuvant reduced bee kills compared with the use of PAM as an adjuvant. This should not be viewed as counterproductive for killing target insect pests in croplands because our prior experiments involving both imidacloprid and bifenthrin with PAM and SA as insecticide adjuvants adequately killed both *L. lineolaris* (tarnished plant bug) and *P. guildinii* (red-banded stink bug)^25^. In fact, both adjuvants exhibited some synergistic activities in killing both insects, which poses significant threats to cotton and soybean in the region^25^.

Our prior experiments with SA have proven that it (a) is compatible with commonly used herbicides and insecticides, (b) does not interfere with pesticide mechanisms to kill target crop pests, and (c) results in a reduction in off-target drift when it is added to herbicides as a drift-reducing adjuvant. This study revealed that SA does not have any aggravated effects on beneficial insects, such as honeybees. To confirm the toxicity results obtained in this study additional experiments will be carried out. Oral exposure through contaminated nectar, pollen and water is another route of pesticide exposure to honeybees. It affects the gut microbiome, feeding behavior, detoxification enzyme activity, and long-term survival. Therefore, we are currently investigating oral pesticide exposure to honeybees as well to fully validate SA on toxicity. Sublethal effects of pesticide exposure that affects impaired foraging, learning and immune suppression will also be investigated in the future.

The literature has shown that the pesticide adjuvants presently in use are toxic to pollinators. The gap in the literature is the identification of alternative materials as potential pesticide adjuvants, which the current study focused on. The results from this study, along with the results from our prior studies, collectively indicate that SA could be a pollinator-friendly drift reducing pesticide adjuvant. SA as a pesticide adjuvant has a considerable positive effect on the pesticide market. Additionally, SA deserves to receive encouragement from redefined policies on the use of adjuvants for pesticide application.

## Conclusions

The interactions of sodium alginate (SA, an adjuvant) with two commonly used insecticides (imidacloprid and bifenthrin) were examined for the first time in honeybees. Neither the water alone nor the adjuvants alone killed the honeybees. The finding of antagonistically reduced bee mortality (compared with the standard insecticide adjuvant PAM) from this study, together with our previous findings of drift reduction and synergistic action against the tarnished plant bug and the red-banded stink bug, consistently demonstrated that SA could be a pollinator-friendly drift-reducing pesticide adjuvant. The use of adjuvants such as SA could lead to paradigm shifts in the use of pesticide adjuvants to kill target pests and protect beneficial insects simultaneously.

## Electronic supplementary material

Below is the link to the electronic supplementary material.

Supplementary Material 1

## Data Availability

The honeybee toxicity data generated during the current study are not publicly available but are available from the corresponding author on reasonable request.
