# Supplementary material for: Investigation of an algae-derived polymer as a pollinator-friendly pesticide adjuvant
Source: Sci Rep. 2025 Jul 15;15:25559. doi: 10.1038/s41598-025-10558-1 (PMC12264170; doi:10.1038/s41598-025-10558-1)
Supplement: Supplementary file 1 — Supplementary Material 1 [file 41598_2025_10558_MOESM1_ESM.docx]

Investigation of an algae-derived polymer

as a pollinator-friendly pesticide adjuvant

**Narayanan Kannan^1,*^ and Yu-Cheng Zhu^2^**

***Supplementary material***

**Table S1:** Results of corrected mortality for different treatments

| **Treatments** | **Corrected Mortality**  **(%±SE)** | **Letter indicating difference*** |
| --- | --- | --- |
| A1.25 + I483  I483  P150 + I483  P75 + I483  A2.5 + I483  P150 + I276  I276  P75 + I276  A1.25 + I276  A2.5 + I276  P75 + I158  I158  A1.25 + I158  P150 + I158  B 446  A2.5 + I158  P75 + B446  B150  P150 + B258  A1.25 + B446  P150 + B446  B258  P75 + B258  P75 + B150  A2.5 + B446  A1.25 + B258  A1.25  P150  A1.25 + B150  A2.5 + B150  A2.5 + B258  A 2.5  P150 + B150  P75 | 100±0  100±0  100±0  100±0  98.31±1.69  96.61±1.69  96.61±3.39  96.61±3.39  94.92±2.94  91.53±3.39  91.53±4.48  84.75±5.87  83.05±3.39  81.36±1.7  77.97±9.44  72.88±13.24  66.10±16.69  49.15±22.16  32.20±17.2  30.51±8.97  30.51±17.94  30.51±24.97  27.12±3.39  22.03±11.12  20.34±4.48  6.78±4.49  5.86±3.8  0.00±1.7  -1.7±0  -1.7±0  -1.7±0  -1.7±0  -1.7±0  -1.7±0 | a  a  a  a  a  ab  ab  ab  ab  ab  ab  abc  abc  abc  abc  bcd  cd  de  ef  efg  efg  efg  efgh  fghi  fghi  ghi  hi  i  i  i  i  i  i  i |

*Means followed by different letters are significantly different (F(34)=22.23, P<0.001)

**Table S2:** Results of t Test (LSD) for corrected mortality

| **Comparison between** | | **Difference between means** | **95% confidence limits** | |
| --- | --- | --- | --- | --- |
| **Treatment 1** | **Treatment 2** |  | **Lower** | **Upper** |
| P+I  P+I  P+I  P+I  P+I  P+I  P+I  P+I  I  I  I  I  I  I  I  I  A+I  A+I  A+I  A+I  A+I  A+I  A+I  A+I  B  B  B  B  B  B  B  B  P+B  P+B  P+B  P+B  P+B  P+B  P+B  P+B  A+B  A+B  A+B  A+B  A+B  A+B  A+B  A+B  A  A  A  A  A  A  A  A  W  W  W  W  W  W  W  W  P  P  P  P  P  P  P  P | I  A+I  B  P+B  A+B  A  W  P  P+I  A+I  B  P+B  A+B  A  W  P  P+I  I  B  P+B  A+B  A  W  P  P+I  I  A+I  P+B  A+B  A  W  P  P+I  I  A+I  B  A+B  A  W  P  P+I  I  A+I  B  P+B  A  W  P  P+I  I  A+I  B  P+B  A+B  W  P  P+I  I  A+I  B  P+B  A+B  A  P  P+I  I  A+I  B  P+B  A+B  A  W | 0.564  4.237  41.809^*^  64.973^*^  85.597^*^  92.271^*^  94.354^*^  95.203^*^  -0.564  3.672  41.244^*^  64.408^*^  85.033^*^  91.707^*^  93.79^*^  94.638^*^  -4.237  -3.672  37.572^*^  60.736^*^  81.361^*^  88.034^*^  90.118^*^  90.966^*^  -41.809^*^  -41.244^*^  -37.572^*^  23.164^*^  43.788^*^  50.462^*^  52.546^*^  53.394^*^  -64.973^*^  -64.408^*^  -60.736^*^  -23.164^*^  20.624^*^  27.298^*^  29.382^*^  30.23^*^  -85.597^*^  -85.033^*^  -81.361^*^  -43.788^*^  -20.624^*^  6.674  8.757  9.606  -92.271^*^  -91.707^*^  -88.034^*^  -50.462^*^  -27.298^*^  -6.674  2.083  2.932  -94.354^*^  -93.79^*^  -90.118^*^  -52.546^*^  -29.382^*^  -8.757  -2.083  0.848  -95.203^*^  -94.638^*^  -90.966^*^  -53.394^*^  -30.23^*^  -9.606  -2.932  -0.848 | -14.288  -7.89  26.956  52.846  73.47  75.121  71.667  78.052  -15.417  -11.18  24.094  49.556  70.18  72.532  69.536  75.464  -16.364  -18.525  22.72  48.609  69.233  70.884  67.43  73.816  -56.662  -58.395  -52.425  8.311  28.936  31.288  28.291  34.219  -77.1  -79.261  -72.863  -38.017  8.497  10.148  6.694  13.08  -97.724  -99.885  -93.488  -58.641  -32.752  -10.476  -13.931  -7.545  -109.421  -110.881  -105.185  -69.637  -44.449  -23.824  -23.642  -18.073  -117.042  -118.044  -112.806  -76.8  -52.069  -31.445  -27.809  -24.877  -112.353  -113.813  -108.116  -72.569  -47.38  -26.756  -23.936  -26.574 | 15.417  16.364  56.662  77.1  97.724  109.421  117.042  112.353  14.288  18.525  58.395  79.261  99.885  110.881  118.044  113.813  7.89  11.18  52.425  72.863  93.488  105.185  112.806  108.116  -26.956  -24.094  -22.72  38.017  58.641  69.637  76.8  72.569  -52.846  -49.556  -48.609  -8.311  32.752  44.449  52.069  47.38  -73.47  -70.18  -69.233  -28.936  -8.497  23.824  31.445  26.756  -75.121  -72.532  -70.884  -31.288  -10.148  10.476  27.809  23.936  -71.667  -69.536  -67.43  -28.291  -6.694  13.931  23.642  26.574  -78.052  -75.464  -73.816  -34.219  -13.08  7.545  18.073  24.877 |

**Note:** Comparisons significant at the 0.05 level are indicated by *

Alpha 0.05 P: Polyacrylamide

Error Degrees of Freedom 96 I: Imidacloprid

Error Mean Square 335.9255 A: Sodium Alginate

Critical Value of t 1.98498 B: Bifenthrin

W: Water

**Table S3**: Summary statistics for the GLM procedure

| **Source** | **Degree of freedom** | **Sum of squares** | **Mean Square** | **F value** | **P>F** |
| --- | --- | --- | --- | --- | --- |
| Model  Error  Corrected total | 8  96  104 | 156,693.6^*^  32,248.8  188,942.5 | 19,586.7  335.9 | 58.31 | <0.0001 |

^*^Same as type I and Type III sum of squares
